# OD04 The EQ-5D-5L Value Set For Ghana

**DOI:** 10.1017/S0266462324001417

**Published:** 2025-01-07

**Authors:** Rebecca Addo, Brendan Mulhern, Richard Norman, Richmond Owusu, Rosalie Viney, Justice Nonvignon

## Abstract

**Introduction:**

Ghana’s reference case, developed to guide the conduct of economic evaluation as part of health technology assessment (HTA) guidelines, recommends the conduct of cost–utility analysis using outcomes such as quality-adjusted life years (QALYs). There is no national value set available for the Ghanaian population to be used in estimating QALYs. This study aimed to develop a value set for Ghana using the EuroQol 5-dimension 5-level questionnaire (EQ-5D-5L) instrument.

**Methods:**

Face-to-face preference data were collected from 300 adults across three regions of Ghana using the adapted version of the EuroQol Valuation Technology (EQ-VT) standardized valuation protocol developed specifically for EQ-5D-5L valuation studies using composite time-trade-off (cTTO) and discrete choice experiments (DCEs). Different preference models were generated using both the cTTO and DCE data, individually or together to provide complementary results on respondents’ utility preferences. Models explored include generalized least squares, tobit, heteroskedastic, logit, and hybrid. The best-fitting model was selected for the value set based on its logical consistency, ability to account for left-censored and heteroskedasticity data, and statistical significance of parameters.

**Results:**

The 300 interviews provided 4,500 cTTO responses and 4,200 DCE responses. The demographic characteristics of respondents were representative of the Ghanaian population for religious background, level of education, and marital status. The preferred model chosen for the Ghana value set was hybrid tobit, random effect heteroskedastic, constrained model. The predicted value for the worst attainable health on the EQ-5D-5L (i.e., health state 55555) was −0.493 and that of the best health state (11112; except full health) was 0.969. The largest decrement was registered for level five mobility (0.369) followed by pain/discomfort (0.312), self-care (0.273), anxiety/depression (0.271), and usual activities (0.268).

**Conclusions:**

This is the first Ghanaian EQ-5D-5L value set based on social preference derived for a nationally representative sample in Ghana. The value set will play a key role in the institutionalization of HTA in Ghana and the use of economic evaluation studies to inform priority setting where different health technologies can be compared. A planned findings dissemination to stakeholders is underway.

